# ALSDet: a global context-enhanced network for detecting small-target diseases on apple leaves

**DOI:** 10.3389/fpls.2026.1883033

**Published:** 2026-08-07

**Authors:** Xulu Gong, Pengfei Xu, Rui Ren, Fuping Gong, Shujuan Zhang

**Affiliations:** 1 Faculty of Software Technologies, Shanxi Agricultural University, Jinzhong, China; 2 School of Mathematical Sciences, Shanxi Normal University, Taiyuan, China; 3 College of Agricultural Engineering, Shanxi Agricultural University, Jinzhong, China; 4 Linfen Meteorology Bureau, Linfen, China

**Keywords:** apple leaf diseases, convolutional neural network, global context, object detection, small target

## Abstract

Accurate detection of small-target diseases on apple leaves is of great importance for optimizing orchard management and facilitating precision agriculture. Small-target disease detection remains challenging due to insufficient features and complex backgrounds. This work proposes an effective detector for apple leaf small-target diseases called ALSDet. The global context module is integrated into Stage2 to Stage4 of the ResNet-50 backbone, yielding a refinement of the feature-extraction architecture. In the bottleneck blocks of the Stage2 and Stage3, dilated convolution is used in place of the normal 3×3 convolution to enlarge the receptive field for small targets and strengthen feature extraction. During the model training phase, a multi-scale training strategy combined with the online hard example mining method is adopted to focus on learning hard samples and enhance adaptability for various scale targets. According to the experimental results, ALSDet obtains a mean average precision (mAP) of 65.6% and an average recall (AR) of 71.2% on the dataset. The proposed model achieves the highest levels in both mAP and AR when compared to the popular object detection models, such as Cascade R-CNN, Faster R-CNN, GFL, Grid R-CNN, Libra R-CNN, FCOS, VFNet, RetinaNet, SSD, YOLOv7, and YOLOv8. For small-target diseases like rust and frog eye leaf spot, the average precision surpasses 87% with an intersection over union (IoU) threshold of 0.5. These results confirm that ALSDet achieves stable performance against existing methods, demonstrating its potential as a practical tool for intelligent orchard disease management.

## 1 Introduction

Object detection algorithms have been significantly improved by recent advances in deep learning (Sharma and Mir, 2020; Zou et al., 2023; Wu et al., 2020). These methods primarily focus on large targets characterized by distinct contours and rich features, but only a few are designed to detect small objects (Liu et al., 2021), especially small disease lesions on apple leaves. Since timely identification of such small lesions is pivotal to mitigating substantial crop yield losses (Xie et al., 2024; Sunil et al., 2023), developing accurate and efficient detection frameworks has become an urgent unmet demand in precision crop protection.

In MS-COCO, small objects are defined as those with an area of less than 32 by 32 pixels (Lin et al., 2014). Detecting these objects is considerably more challenging than identifying medium and large ones. This difficulty stems primarily from the low resolution and insufficient features of small objects, as well as the scarcity of samples and class imbalance (Tong and Wu, 2022). In recent years, small object detection has emerged as one of the most significant challenges in computer vision.

Remarkably, robust small object detection is vital for numerous practical applications, involving pedestrian detection (Liu et al., 2019), traffic sign recognition (Liu et al., 2020), face detection (Zhang et al., 2020), remote sensing target detection (Ding et al., 2022), agricultural targets detection (Zhao et al., 2025c; Li et al., 2025; You et al., 2025; Liu et al., 2026; Zhao et al., 2025b) and plant disease detection (Gao et al., 2024; Qiao et al., 2025; Ye et al., 2024; Yao et al., 2024; Li et al., 2022). To address these demands, convolutional neural networks (CNNs) (LeCun et al., 1998) have been established as a fundamental deep learning approach for these tasks. Architectures built upon this paradigm—such as the two-stage Faster R-CNN (Ren et al., 2017) and the single-stage YOLO (You Only Look Once) (Sapkota et al., 2025) family—commonly incorporate specialized modules to improve localization and recognition accuracy. An overview of application of CNNs in crop disease detection reveals that multi-scale feature fusion plays an important role in small-target diseases identification. For instance, a YOLOv5-EFFICIENT model, which incorporated an improved spatial pyramid fast fusion module, was developed for rice leaf disease recognition (Gao et al., 2024). Qiao et al. (2025) proposed an improved YOLOv5s for the accurate detection of cucumber downy mildew spores in microscopic images. An alternative strategy frequently employed for identifying small-target diseases involves expanding the receptive field while maintaining resolution using sophisticated convolutional architectures. In YOLOv8, traditional convolutions have been replaced with variable kernel convolutions to enable multi-scale object sampling (Ye et al., 2024). Beyond architectural changes, loss function optimization has also proven effective in recognizing small-target diseases (Yao et al., 2024). Furthermore, multi-scale training and testing strategies can directly benefit deep CNN-based small object detectors. Li et al. (2022) introduced a cucumber disease detection approach that combines multi-scale training with a feature fusion network. In their experimental validation, this strategy effectively enhanced identification performance on tiny disease spots.

Recently, deep learning has been extensively applied to various agricultural vision tasks, demonstrating remarkable progress in crop monitoring, weed management, and horticultural object detection. For instance, Zhao et al. (2025a) proposed a UAV-assisted framework integrating super-resolution reconstruction with semantic segmentation for blueberry maturity monitoring, achieving an mIoU of 83.15% using Mamba-based architectures. Similarly, Tao et al. (2025) combined Transformer and Mamba models for automatic segmentation of weeds and crops in UAV imagery, reporting an mIoU of 90.75% on tobacco field datasets. On the detection front, a series of task-specific YOLO variants have been developed for diverse agricultural targets. Zhao et al. (2025c) introduced Mussel-YOLO for freshwater mussel detection in turbid underwater environments, achieving an mAP of 91.4%. Li et al. (2025) developed Succulent-YOLO for UAV-assisted succulent plant monitoring, reaching an mAP of 87.8%. Multiple YOLO-based detectors have been customized for rose detection (You et al., 2025; Liu et al., 2026; Zhao et al., 2025b). However, they predominantly focus on macro-level targets such as fruits, flowers, weeds, and entire plants, often captured under relatively controlled or UAV-based imaging conditions. In contrast, the detection of early-stage pathological lesions on plant leaves presents fundamentally different challenges.

Among these, apple leaf disease has drawn particular attention for its economic importance and challenging orchard diagnosis. Scholars have achieved efficient and accurate disease identification by adopting various object detection methods. Hou et al. (2023) constructed a novel detection framework by integrating feature pyramid networks, inception module, and squeeze-and-excitation mechanism into ResNet-Faster RCNN. The proposed model attained an average precision of 62.71%. To enhance mobile deployment, Sun et al. (2021) developed a lightweight detection algorithm named MEAN-SSD, which incorporates the MEAN block and Apple-Inception module to realize real-time inference in practical applications. Similarly, building upon the SSD framework, Tian et al. (2023) developed a v-space-guided multi-scale feature fusion model termed VMF-SSD, which enables more robust representation learning toward disease spots with diverse scales. In the meanwhile, efforts were also made towards developing lightweight models, such as the apple leaf disease detection based on YOLOv5s (Xu and Wang, 2023), and the FSM-YOLO based on YOLOv8 (Yan and Yang, 2024). Additionally, a comprehensive deep learning-based system for apple disease recognition was proposed (Khan et al., 2022). The system operates in a two-stage pipeline: a lightweight classification and the detection. In the aforementioned studies, deep learning-based apple leaf disease detection models have demonstrated high accuracy on specific datasets.

However, most of these datasets contain images with relatively simple backgrounds, failing to adequately represent complex field conditions. This limitation restricts the generalization ability of the proposed models to real agricultural scenarios. More importantly, the challenge of small-target diseases detection has been largely overlooked. Small lesions, despite their critical importance for early-stage diagnosis, are often poorly detected. Meanwhile, numerical analysis specifically focused on small-target detection is still relatively scarce. Few studies provide quantitative evaluations of small lesion detection performance, making it difficult to benchmark progress in this area. Therefore, constructing a model capable of addressing the detection of small lesions in natural environments remains a noteworthy and persistent challenge.

This work presents a novel detector termed ALSDet, specifically designed for identifying small lesions on apple leaves under natural field conditions. The ALSDet is designed on the basis of Cascade R-CNN model. The core contributions of this paper are outlined below:

1. A global context module is incorporated into the Stage2 to Stage4 of the ResNet-50 backbone network. This module directs the model’s attention to salient image regions, enabling precise extraction of disease-specific features.
2. In Stage1 to Stage4 of ResNet-50, the dilation rates of the 3×3 convolutions within the bottleneck modules of these four units are set to 1, 2, 2, 1, respectively. This configuration expands the receptive field and strengthens the recognition capability of specific targets.
3. To enhance model robustness and generalization, two complementary training strategies are employed. First, online hard example mining (OHEM) is implemented to prioritize challenging samples during training, effectively mitigating overfitting. Second, a multi-scale training strategy is adopted to improve small-object detection by exposing the model to features across diverse spatial scales.

The subsequent sections of this paper are structured as follows. Section 2 describes the employed materials and the proposed ALSDet detector. Section 3 elaborates the experimental results and corresponding analysis, while Section 4 reports the discussion. Section 5 finally concludes the work.

## 2 Materials and methods

### 2.1 Dataset

**Figure 1:**
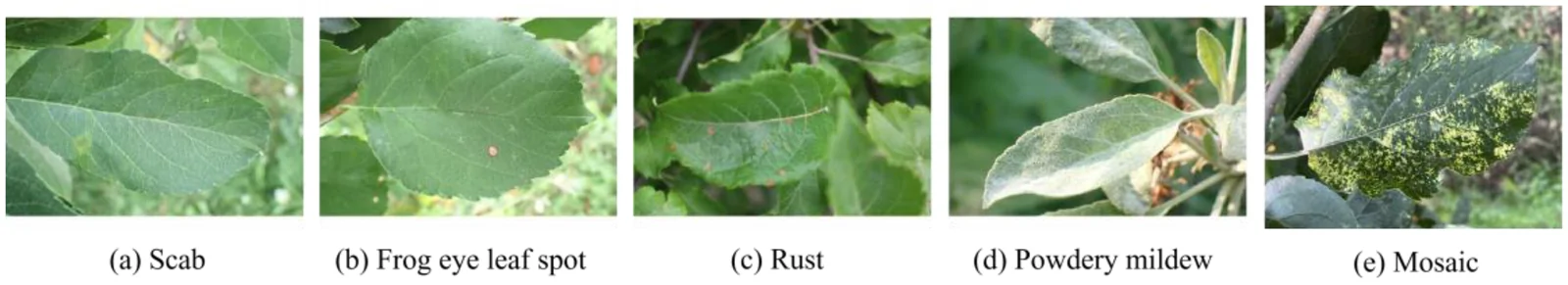
Examples of apple leaf disease images. **(a)** Scab; **(b)** Frog eye leaf spot; **(c)** Rust; **(d)** Powdery mildew; **(e)** Mosaic.

The apple leaf disease dataset utilized in this study is identical to that presented in our previously published work (Gong and Zhang, 2023). The dataset combines a public database and self-collected subset. The open-source data were derived from the CVPR 2021 FGVC8 Plant Pathology Challenge (Thapa et al., 2020) and the open dataset released on Paddle AI Studio. Only apple disease images that satisfied the experimental criteria were retained. The self-collected dataset was acquired from the Pomology Institute of Shanxi Agricultural University and local commercial orchards in Taigu District, Jinzhong City, Shanxi Province. Field image acquisition was conducted from June to August 2022, between 8:00 a.m. and 6:00 p.m. All images were captured using an iPhone 13 with a native camera resolution of 3024×4032 pixels. The shooting distance between the lens and leaf surface was controlled within the range of 10–40 cm. Diversified disease images were obtained by adjusting shooting angles and camera positions during data collection. The final integrated dataset covered five categories of apple leaf diseases, consisting of a total of 4,182 images. Representative samples of each disease type are presented in **Figure 1**. This dataset consists of five categories of apple leaf diseases, including common types scab (*Venturia inaequalis* (Cooke) G. Winter), frog eye leaf spot (*Phyllosticta pirina* Sacc.), rust (*Gymnosporangium asiaticum* Miyabe ex G. Yamada), powdery mildew (*Podosphaera leucotricha* (Ell. and Ev.) Salm.), and mosaic (Apple mosaic virus).

The annotation of apple leaf lesion regions was performed using LabelImg software, with the resulting information—including disease categories and their corresponding spatial locations—stored in XML files associated with each image. The entire dataset was subsequently partitioned by category into training, validation, and test sets at a ratio of 8:1:1. To address the issue of class imbalance, offline data augmentations were specifically applied to the training subset of mosaic disease, including horizontal flips, vertical flips, and random rotations. This augmentation strategy expanded the training set from 3,216 to 3,851 images, with the detailed counts for each type of apple leaf disease presented in **Table 1**. All processes were carried out under the supervision and verification of plant pathology experts.

**Table 1:** Dataset distribution for apple leaf disease.

Disease type	Number of images	Training set	Enhanced training set	Validation set	Test set
Scab	915	733	733	91	91
Frog eye leaf spot	952	762	762	95	95
Rust	928	743	743	93	92
Powdery mildew	903	723	723	90	90
Mosaic	378	190	760	94	94
Total number	4,182	3,216	3,851	484	482

It is worth noting that all apple leaf diseases in this dataset are derived from natural environmental conditions. Notably, the primary distinction between this work and existing studies resides in its algorithmic framework and research emphasis. Whereas previous investigations focused on generic apple leaf disease detection using conventional deep learning architectures, the present study introduces a novel detector, ALSDet, which is specifically designed to identify small-scale apple leaf lesions that are easily overlooked or misclassified in complex orchard scenarios.

### 2.2 The analysis of small target diseases

Within the field of object detection, small objects are typically categorized by two criteria: absolute scale and relative scale. Following the MS-COCO standard, the absolute scale refers to targets smaller than 32×32 pixels. This criterion is intuitive and simple. The relative scale criterion defines small objects according to the ratio of their bounding-box size to the total image area. For each annotated instance, the relative area R is defined as Equation 1.

(1) $$R=\frac{A_{bbox}}{A_{image}}\times 100\%$$

Where $A_{bbox}$ and $A_{image}$ denote the bounding-box area and image area, respectively. For objects within a given category, the median of this ratio typically ranges from 0.08% to 0.58% (Chen et al., 2016). This relative approach aligns more closely with human visual perception, thus providing a more accurate reflection of the target’s actual proportion within the image.

**Table 2** reports both criteria for our dataset. As shown in **Table 2**, the median R ranges from 0.24% to 50.08%. The two categories with the lowest median R, frog eye leaf spot and rust, are visually extremely small and constitute the primary motivation for our ALSDet design. To provide a consistent basis for MS-COCO scale categorization, we uniformly resized all images to 640×640 pixels for statistical analysis. We computed the MS-COCO absolute-scale distribution, which yielded three instance-size categories: 1,494 small instances under 32×32 pixels, 939 medium instances between 32×32 and 96×96 pixels, and 1,278 large instances over 96×96 pixels. Importantly, the two criteria are logically consistent in our dataset. As detailed in **Table 2**, the frog eye leaf spot and rust with the lowest median R contribute all 1,494 MS-COCO-defined small lesion instances. In contrast, powdery mildew, mosaic and scab with high median are predominantly classified as MS-COCO large instances. This cross-verified consistency proves that the relative scale metric reflects the same lesion size trend as the widely adopted MS-COCO absolute standard. Therefore, frog eye leaf spot and rust are the primary targets for small-object detection analysis. Although our dataset is dominated by small targets, it also includes three large target categories, reflecting the natural diversity of disease symptoms in real orchards. This composition allows us to evaluate whether the detector optimized for small targets generalizes effectively to larger ones.

**Table 2:** The calculation results of the two criteria for small targets in the dataset.

Category	Instances	Median R (%)	Small	Medium	Large
Frog eye leaf spot	1,249	0.24	825	419	5
Rust	1,208	0.29	669	514	25
Powdery mildew	416	33.04	0	0	416
Mosaic	383	43.23	0	6	377
Scab	455	50.08	0	0	455

### 2.3 The ALSDet detection model

#### 2.3.1 The baseline network

**Figure 2:**
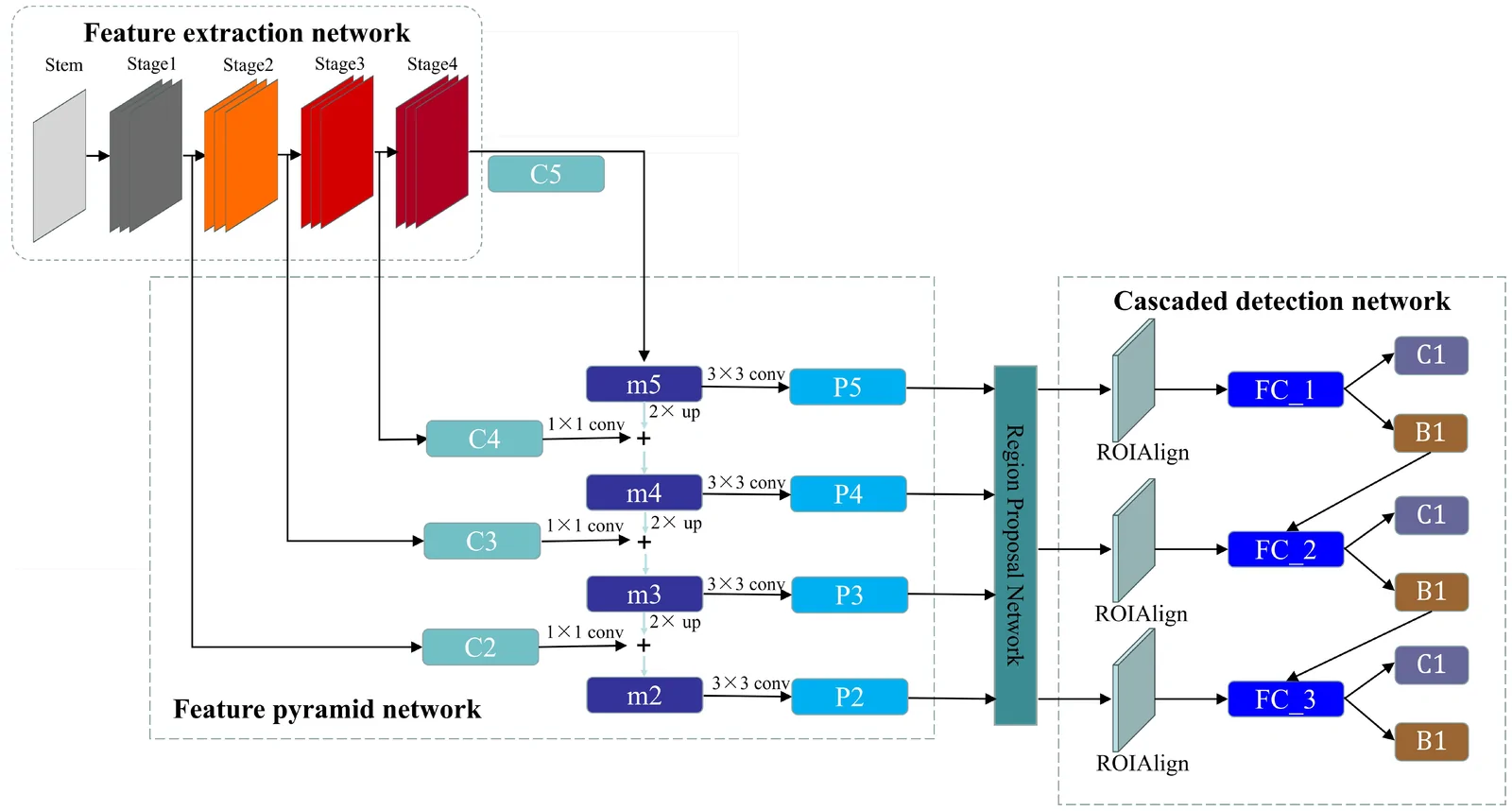
Cascade R-CNN network architecture.

The choice of the intersection over union (IoU) threshold substantially affect detection performance, particularly for small objects where accurate target matching is challenging. To address this issue, a cascade structure with progressively increasing IoU thresholds is introduced by Cascade R-CNN (Cai and Vasconcelos, 2018). This design enables the systematic reselection of positive and negative samples at different stages. Thus, the framework enhances the localization quality of detection boxes and effectively fulfills the demanding requirements of small object detection. Owing to these capabilities, Cascade R-CNN was employed as the foundational framework in this study. The Cascade R-CNN framework, illustrated in **Figure 2**, comprises three main components: a ResNet feature extraction backbone, a feature pyramid network (FPN), and a cascade detection network. During feature extraction, the ResNet backbone extracts deep feature representations from input images. The hierarchical convolutional layers within ResNet, denoted as Stem through Stage4, produce intermediate feature maps C2, C3, C4, and C5. These features are subsequently fused to generate multi-scale feature representations P2, P3, P4, and P5. Subsequently, these multi-scale feature representations are fed into the region proposal network (RPN) to produce high-quality candidate regions. In the detection stage, RoIAlign is first adopted to precisely align each region of interest (RoI) onto a fixed-scale feature map. These feature maps are then used as input data for fully connected layers to compute classification scores C and bounding box regression offsets B. Cascade R-CNN employs a multi-stage cascade architecture, where each stage refines the detection predictions under increasing IoU thresholds. This cascaded mechanism effectively mitigates overfitting during training and alleviates the IoU mismatch issue during inference, thereby significantly enhancing the overall accuracy of object detection.

#### 2.3.2 Global context block

The non-local block (Wang et al., 2018) serves as a representative mechanism for modeling long-range dependencies through information aggregation across all spatial positions in a feature map. Its simplified variant, the simplified non-local (SNL) block, streamlines this process. However, the SNL block still introduces a considerable parameter overhead due to its 1×1 convolution transformation module. To address this limitation, we adopt the global context (GC) block (Cao et al., 2019), which enhances the SNL block by incorporating a bottleneck transformation inspired by the squeeze-excitation (SE) network (Hu et al., 2018).

**Figure 3:**
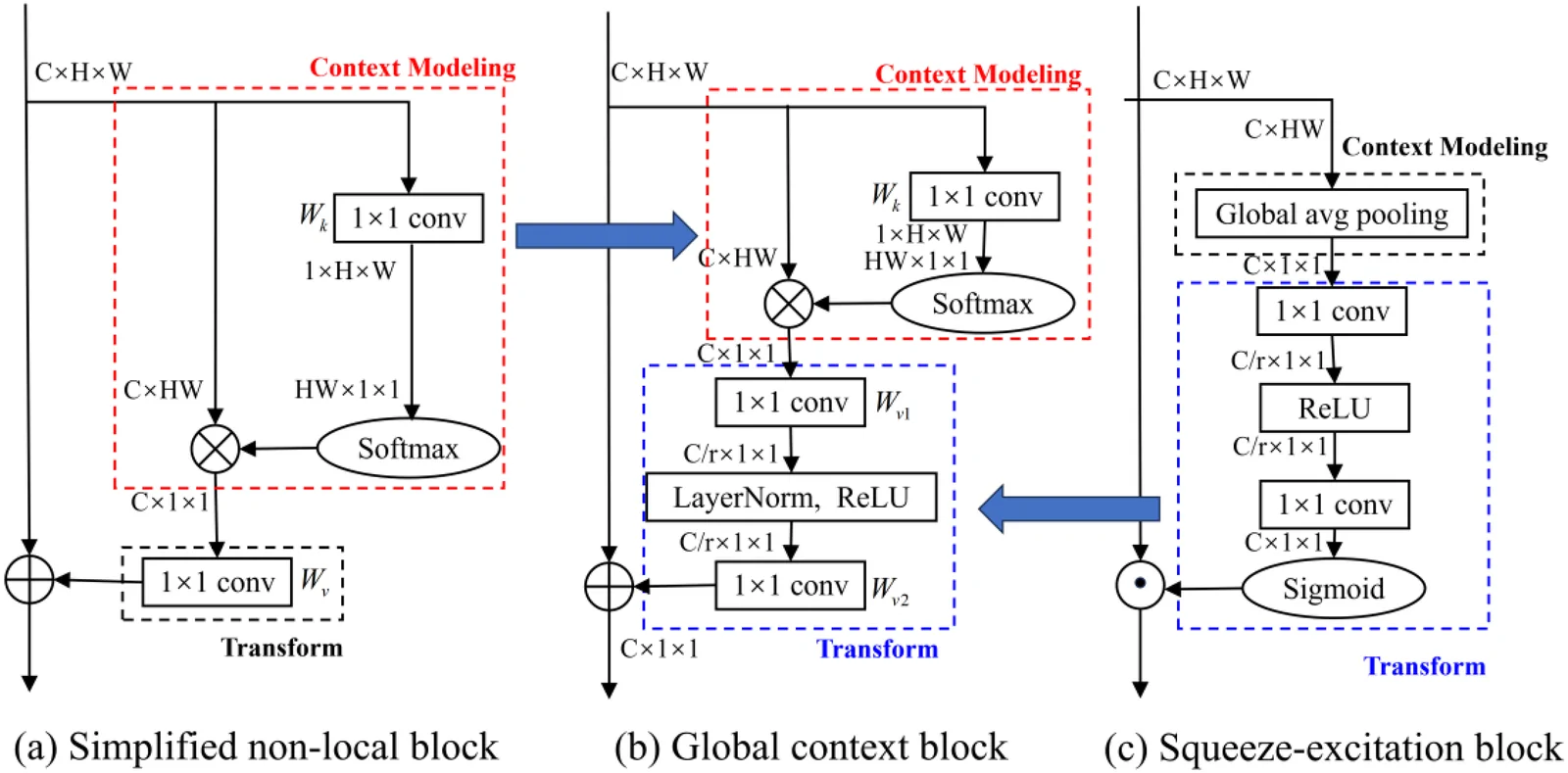
Relationship between the global context block and the simplified non-local/squeeze-excitation block. **(a)** Simplified non-local block; **(b)** Global context block; **(c)** Squeeze-excitation block.

The architectures of the simplified non-local block and squeeze-excitation block are shown in **Figures 3A, C**, respectively. As illustrated in **Figure 3B**, the GC block first employs a context modeling module to aggregate global information, yielding a position-independent global context feature. This feature is subsequently fed into a bottleneck transform module, where the original 1×1 convolution is substituted by a dual-layer bottleneck structure with a reduction ratio r=16. This design reduces the parameter count from C×C to 2×C×C/r. To mitigate the increased optimization difficulty introduced by the bottleneck structure, layer normalization is applied prior to the ReLU activation. Finally, the transformed global context is fused back to the features at each position via addition. The complete operation of the GC block is defined as Equation 2.

(2) zi=xi+Wv2ReLU(LN(Wv1∑j=1NpeWkxj∑m=1NpeWkxmxj))

where $x_{i}$ and $z_{i}$ are the input and output features at position i, $W_{k}$, $W_{v1}$ and $W_{v2}$ are learnable linear transformation matrices.

The GC module combines the advantages of effective context modeling from SNL network and lightweight design from SE network. This characteristic allows for its straightforward deployment within the feature extraction backbone of detectors. In our work, the GC module is incorporated into the Stage2 to Stage4 of the ResNet-50 architecture. Despite incurring minimal additional computational overhead, the network gains the capability to capture long-range spatial relationships and refine its holistic feature representation. Based on this, the model can focus on key features, enhance the discriminability between cluttered backgrounds and target regions, and ultimately improve object detection performance.

#### 2.3.3 Residual module with dilated convolution

In He et al. (2016)’s design, two types of residual modules are proposed: a basic version and a bottleneck structure. The latter, which is designed to reduce computational load, stacks three convolutional layers (1×1, 3×3, 1×1) and incorporates an identity shortcut connection. Typically, the 3×3 convolution in this module is a standard convolution operation. However, this module only focuses on local features, which limits the flexibility of the receptive field. To increase the perceptual area of the standard convolutional filters, dilated convolutions are adopted in this work.

Dilated convolution, also known as atrous convolution, is first introduced by Yu and Koltun (2016) to address challenges in semantic segmentation tasks. Specifically, for a convolutional layer equipped with a k×k kernel and a dilation factor r, the resultant equivalent kernel size is expressed as in Equation 3.

(3) K=k+(k−1)∗(r−1)

The calculation of the receptive field for the initial convolutional layer utilizing dilated convolution is expressed in Equation 4.

(4) $$R_{1}=K_{1}$$

For subsequent convolutional layers, Equation 5 is employed to determine the receptive field.

(5) $$R_{n}=R_{n-1}+\left(K_{n}-1\right)\prod_{i=1}^{n-1}S_{i}$$

where $R_{n}$, $K_{n}$, and $S_{n}$ denote the receptive field, equivalent kernel size, and convolution stride of the n-th layer.

The feature extraction backbone adopted here is ResNet-50. From Equation 5, it can be seen that the receptive field of a standard bottleneck residual block, after three successive convolutions, is limited to 3. To address this limitation and facilitate the detection of small-scale objects through multi-scale feature learning, we introduce dilated convolutions with a rate of 2 into the 3×3 convolutional layers of specific bottleneck blocks. Following this modification, the receptive field of the third layer in such a block increases to 5, representing a notable expansion compared to the original configuration. With dilated convolution, the model gains a larger receptive field and richer multi-scale context, thereby improving the overall detection performance and inference speed.

#### 2.3.4 Improved ResNet-50 backbone network

**Figure 4:**
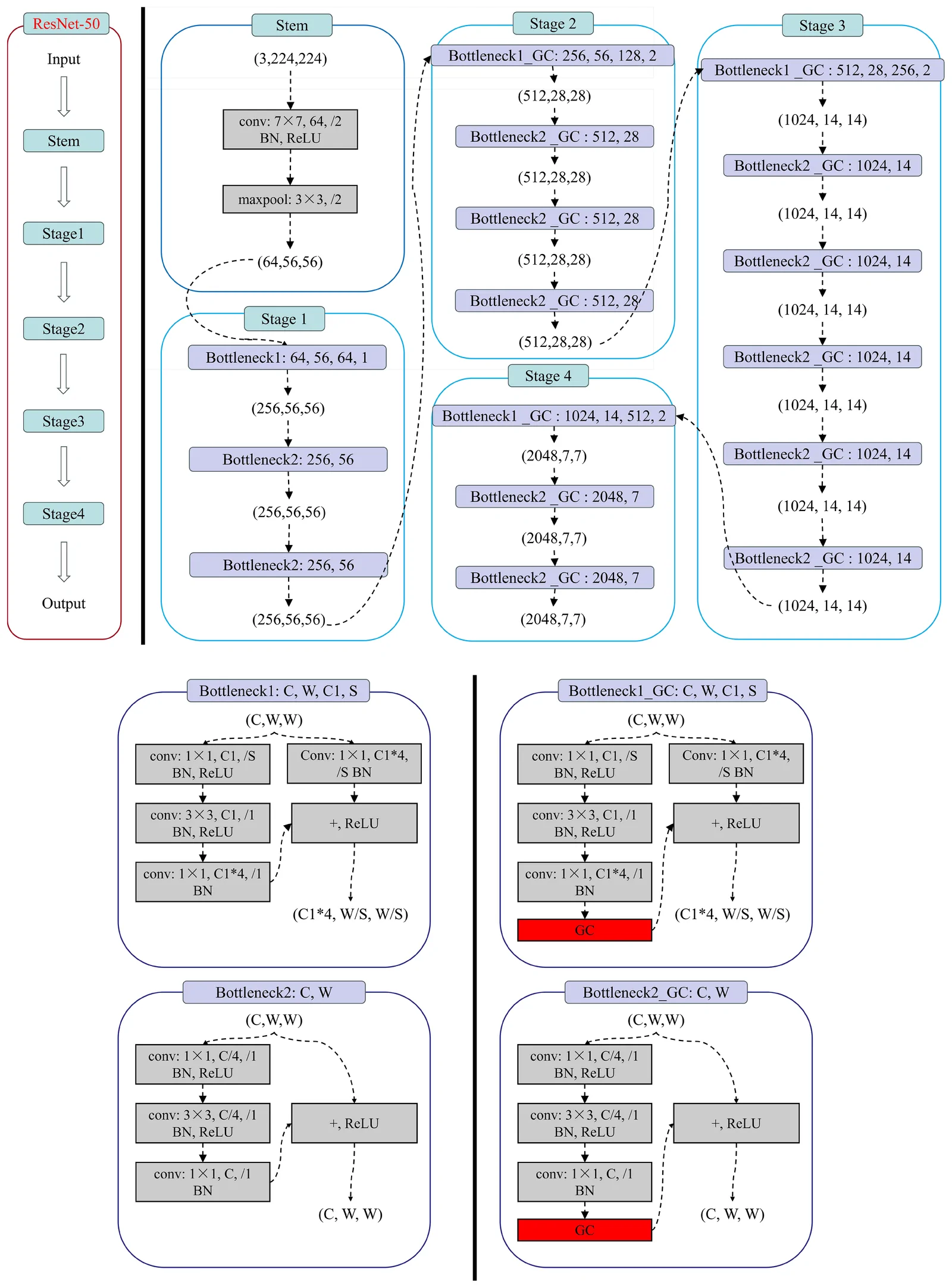
The improved architecture of the ResNet-50 network.

The improved ResNet-50 architecture proposed in ALSDet is illustrated in **Figure 4**. ResNet-50 consists of 50 convolutional layers, which are divided into five units: Stem, Stage1, Stage2, Stage3, and Stage4. Stem is structured with a 7×7 convolutional layer, after which a max-pooling operation is applied. Stage1 to Stage4 are each composed of multiple bottleneck residual units, specifically 3, 4, 6, and 3 in number. In Stage1, the first residual block is denoted as Bottleneck1, while the remaining modules are Bottleneck2. For Stage2 to Stage4, the first residual module in each stage is Bottleneck1_GC, and the rest are Bottleneck2_GC. In the Bottleneck1, the number of output channels is either 4 times or 2 times that of the input channels. When the stride is set to 2, the size of the feature map is halved. In contrast, Bottleneck2 preserves both the number of channels and the feature map dimensions. Bottleneck1_GC and Bottleneck2_GC are modified by embedding the GC module into Bottleneck1 and Bottleneck2, respectively. Additionally, in Stage2 and Stage3, the 3×3 convolutions in the original bottleneck residual blocks are replaced by dilated convolutions with a dilation rate of 2.

### 2.4 Training strategies

Focusing on the detection of small-target diseases in apple leaves, we implemented two distinct training strategies: OHEM and multi-scale training. The implementation details of each are described as follows.

**Figure 5:**
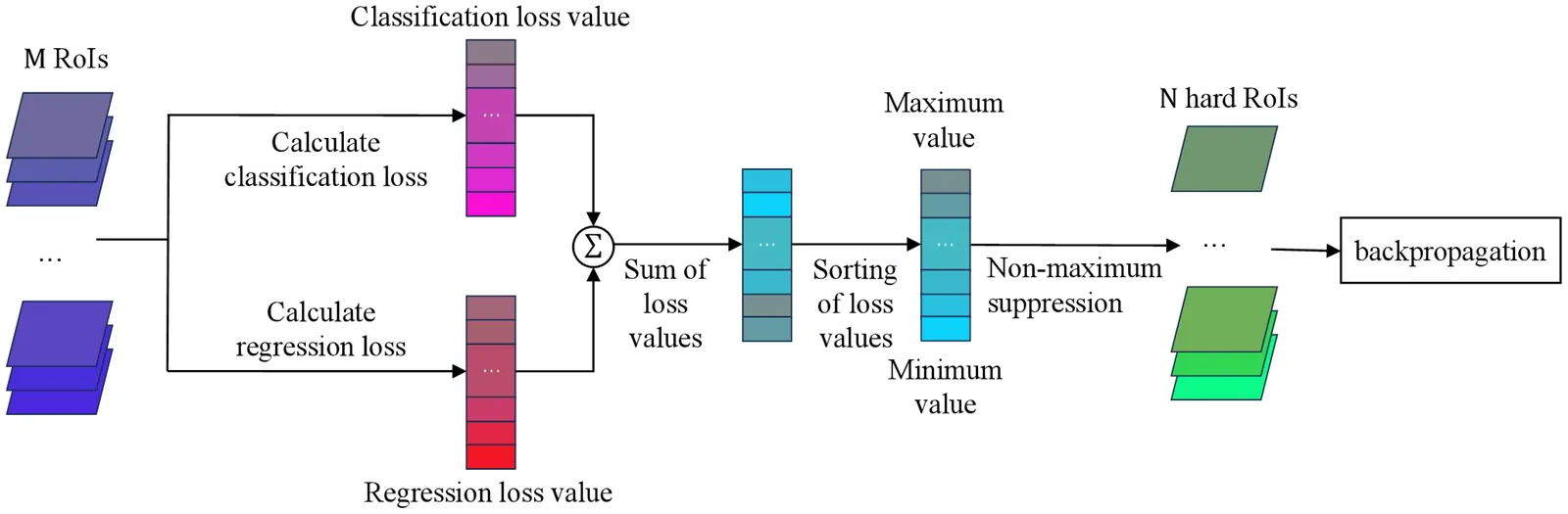
The principle of OHEM.

OHEM addresses the imbalance between hard and easy sample recognition by automatically selecting misclassified examples during the iteration process for training. The principle of OHEM is shown in **Figure 5**. First, a set of M Regions of Interest (RoIs) is acquired via the feature extraction network and the RPN. Next, the classification loss and regression loss for each RoI are computed and summed. These total loss values are subsequently sorted in descending order. Then, RoIs with IoU values greater than the predefined threshold are removed through non-maximum suppression. Finally, the top-N RoIs with the largest total loss values are selected as hard examples to perform backpropagation for parameter updating.

The adopted multi-scale training strategy operates as follows: the input image was first resized to one of the specific scales, i.e., 1333×640, 1333×672, 1333×704, 1333×736, 1333×768, and 1333×800, before being fed into the network for training. These resolutions serve as data augmentation techniques to enhance the model’s scale invariance. This approach not only avoids excessive computational cost but also enhances the model’s adaptability to variations in data size. Consequently, the model can easily learn more abundant features, which improves its reliability and stability across different scenarios.

## 3 Experimental results

### 3.1 Experimental setup

The experimental setup consisted of a workstation operating on Ubuntu 18.04 LTS (64-bit), equipped with an AMD Ryzen 5 5600X CPU (6 cores, 12 threads, base frequency 3.7 GHz), an NVIDIA GeForce RTX 3060 Lite Hash Rate GPU, and 16 GB of RAM. The programming language was Python 3.9.1, the deep learning framework was PyTorch 1.13, and GPU-based computation was enabled via CUDA 11.3 and cuDNN 7.6.

### 3.2 Implementation details

Our model employs an enhanced ResNet-50 backbone network. The improved architecture of the ResNet-50 network is shown in **Figure 4**. The GC block is inserted into all bottleneck blocks within Stage2, Stage3, and Stage4. Regarding the dilation configuration, we applied a dilation rate of 2 to all bottleneck blocks in Stage2 and Stage3, while Stage4 uses a dilation rate of 1. The improved ResNet-50 backbone was initialized with ImageNet-1K pretrained weights.

We trained our ALSDet model using MMDetection for 12 epochs with SGD, using an initial learning rate of 0.02, momentum of 0.9, and weight decay of 0.0001, combined with a linear warmup for 500 iterations and multi-step decay at epochs 8 and 11. Multi-scale training was performed with the short image side randomly sampled from {640, 672, 704, 736, 768, 800} pixels and the long side capped at 1333 pixels, coupled with random horizontal flipping at a probability of 0.5. NMS thresholds were 0.7 for RPN proposals and 0.5 for final detections, with a score threshold of 0.05. All experiments were conducted on a single GPU with a batch size of 2.

### 3.3 Evaluation metrics

Mean Average Precision (mAP) is adopted as the main evaluation metric for object detection, with its computation following Equation 6.

(6) $$\text{m}AP=\frac{1}{N}\sum_{i=1}^{N}AP_{i}$$

Where $AP_{i}$ and Ndenote the i-th category’s average precision and the number of categories, respectively. The definition of AP is provided in Equation 7.

(7) $$AP=\int_{0}^{1}PdR$$

where the symbols P and R stand for precision and recall, respectively, whose mathematical expressions are given in Equations 8 and 9.

(8) $$P=\frac{TP}{TP+FP}$$

(9) $$R=\frac{TP}{TP+FN}$$

In these expressions, TP, FP and FN represent the counts of true positives, false positives, and false negatives.

Based on the setting of IoU thresholds, mAP can be categorized as follows. The primary metric, mAP@[0.5:0.95], is computed as the mean mAP over 10 IoU thresholds between 0.5 and 0.95 with a step of 0.05, constituting the standard benchmark on the MS-COCO dataset. In this study, mAP is uniformly used to represent mAP@[0.5:0.95]. For comprehensive evaluation, we also report results at fixed IoU thresholds of 0.5 and 0.75, corresponding to mAP@0.5 and mAP@0.75. Similarly, average recall (AR) represents AR@[0.5:0.95]. AP_S_, AP_M_, and AP_L_ serve as the metrics for assessing detection performance on small, medium, and large objects, respectively. Model complexity is also assessed via parameter count and floating-point operations (FLOPs).

### 3.4 GC module validation comparison

In ALSDet, the GC module is incorporated into the bottleneck residual blocks, namely Bottleneck1 and Bottleneck2, yielding the newly designed Bottleneck1_GC and Bottleneck2_GC. To evaluate the impact of replacing these new modules either individually in Stage2, Stage3, or Stage4 or comprehensively in all stages from Stage2 to Stage4, a comparative experiment was conducted against the baseline Cascade R-CNN network on the validation set. As illustrated in **Table 3**, all four strategies outperform the baseline across mAP, mAP@0.5, and mAP@0.75. This improvement substantiates that the GC module facilitates the modeling of long-range contextual dependencies, thereby contributing to enhanced detection precision. Despite the slight increase in average detection time, the strategy of deploying the Bottleneck1_GC and Bottleneck2_GC throughout Stage2 to Stage4 in ResNet-50 is adopted, resulting in an improved ResNet-50 backbone architecture, as illustrated in **Figure 4**. This approach provides the best accuracy, resulting in the highest detection accuracy.

**Table 3:** Performance comparison of different replacing methods.

Replacing stage	mAP (%)	mAP@0.5 (%)	mAP@0.75 (%)	Average detection time (s)
Cascade R-CNN	62.0	91.1	68.8	0.077
Stage2	63.4	91.5	69.3	0.079
Stage3	63.2	91.3	69.0	0.079
Stage4	62.4	91.4	68.6	0.078
Stage2 to Stage4	63.5	91.6	69.3	0.083

**Figure 6:**
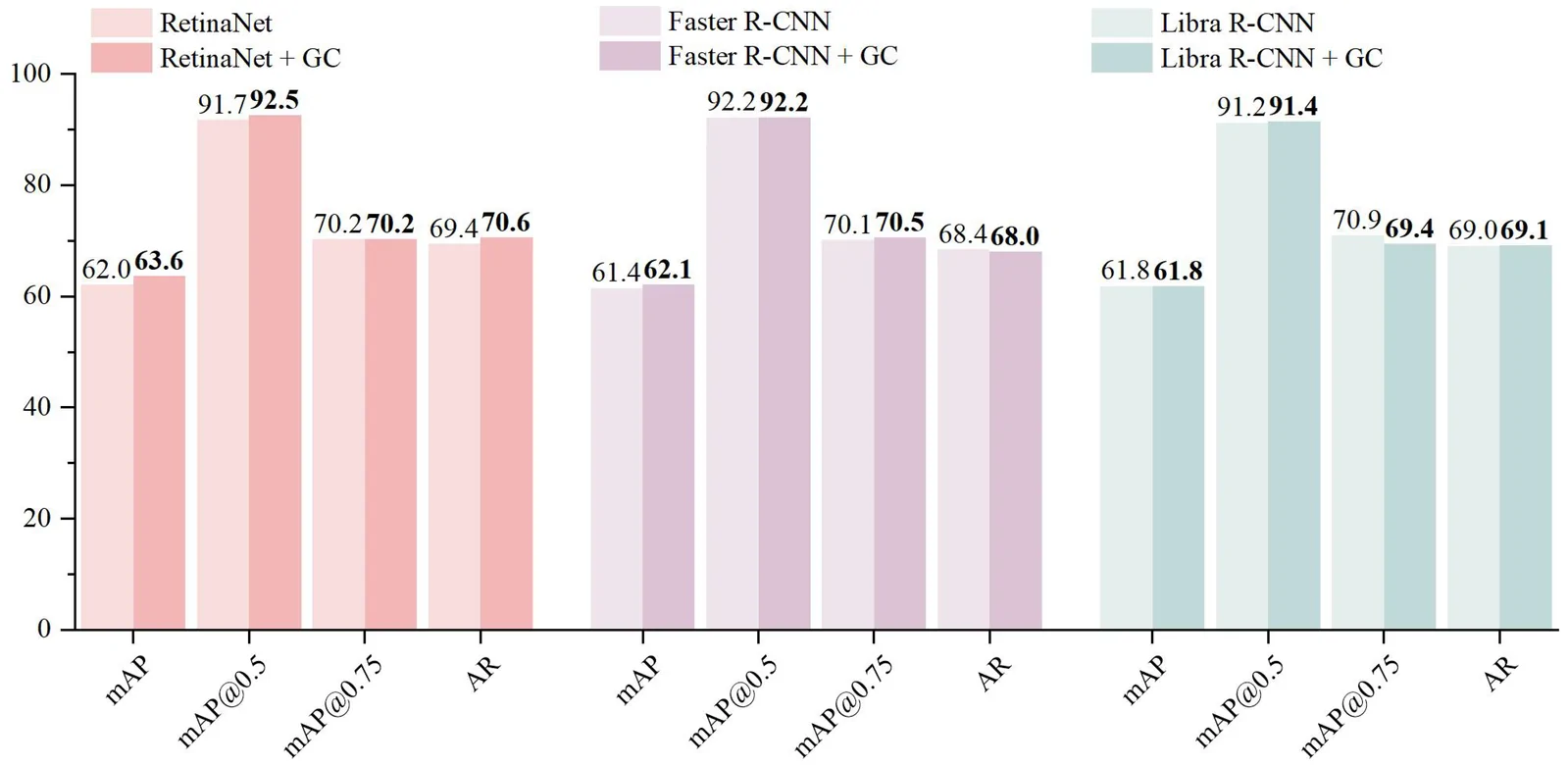
Performance comparison of original and improved ResNet-50 as backbones. (%).

To further validate the efficacy of GC-enhanced ResNet-50, we applied it to three typical detectors and compared its performance with the original backbone in **Figure 6**, where mAP, mAP@0.5, mAP@0.75 and AR are reported. It can be observed that the improved ResNet-50 backbone, which is the ResNet-50 network augmented with the GC module, improves both precision and recall of the model. Among these methods, Libra R-CNN maintains comparable mAP with the improved ResNet-50, accompanied by a 0.2% rise in mAP@0.5 and a 0.1% gain in AR, yet a 1.5% drop in mAP@0.75. This arises from the intrinsic architecture of Libra R-CNN, which is already highly optimized for handling data imbalance, thus leaving limited room for further improvement from the backbone architecture. In conclusion, the GC module robustly enhances feature representation via non-local operations, improving detection accuracy and reducing error rates in various networks.

**Figure 7:**
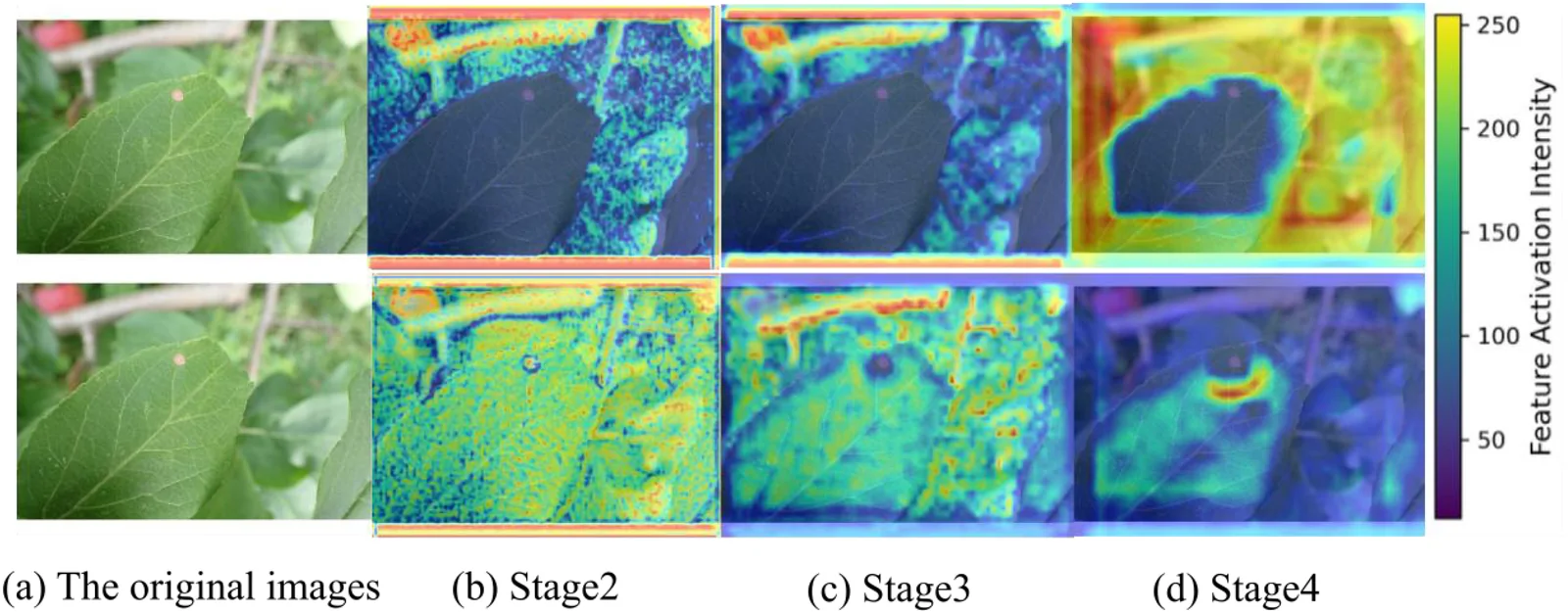
Heatmap comparison between the original Cascade R-CNN and Cascade R-CNN using the improved ResNet-50 after Stage2, Stage3, and Stage4. **(a)** The original images; **(b)** Stage2; **(c)** Stage3; **(d)** Stage4.

Further analysis was performed to investigate the impact of the GC module on the feature learning process of Cascade R-CNN. We compared the feature maps generated by the baseline Cascade R-CNN and the version integrated with our improved ResNet-50, under identical conditions using diseased apple leaf images. **Figure 7** presents the heatmaps of these two methods after the Stage2, Stage3, and Stage4. Warmer colors indicate higher activation intensity, corresponding to regions that contribute most to the disease classification decision. The color bar on the right indicates the normalized activation intensity range, where lower values in blue represent low attention and higher values in red represent high attention. As shown in **Figure 7**, we observe that the original Cascade R-CNN failed to extract disease information accurately and efficiently. It exhibited excessive focus on irrelevant background regions. In contrast, as the network depth increases, the activation in the Cascade R-CNN model utilizing the improved ResNet-50 progressively shifts from scattered edge responses to precise, lesion-focused localization, demonstrating that ALSDet effectively learns to attend to disease-relevant regions. These results demonstrate that, relative to the original Cascade R-CNN, the GC module empowers the model to allocate more attention to category prediction regions and attenuates interference from irrelevant background information.

### 3.5 Dilation rate configurations performance comparison

**Figure 8:**
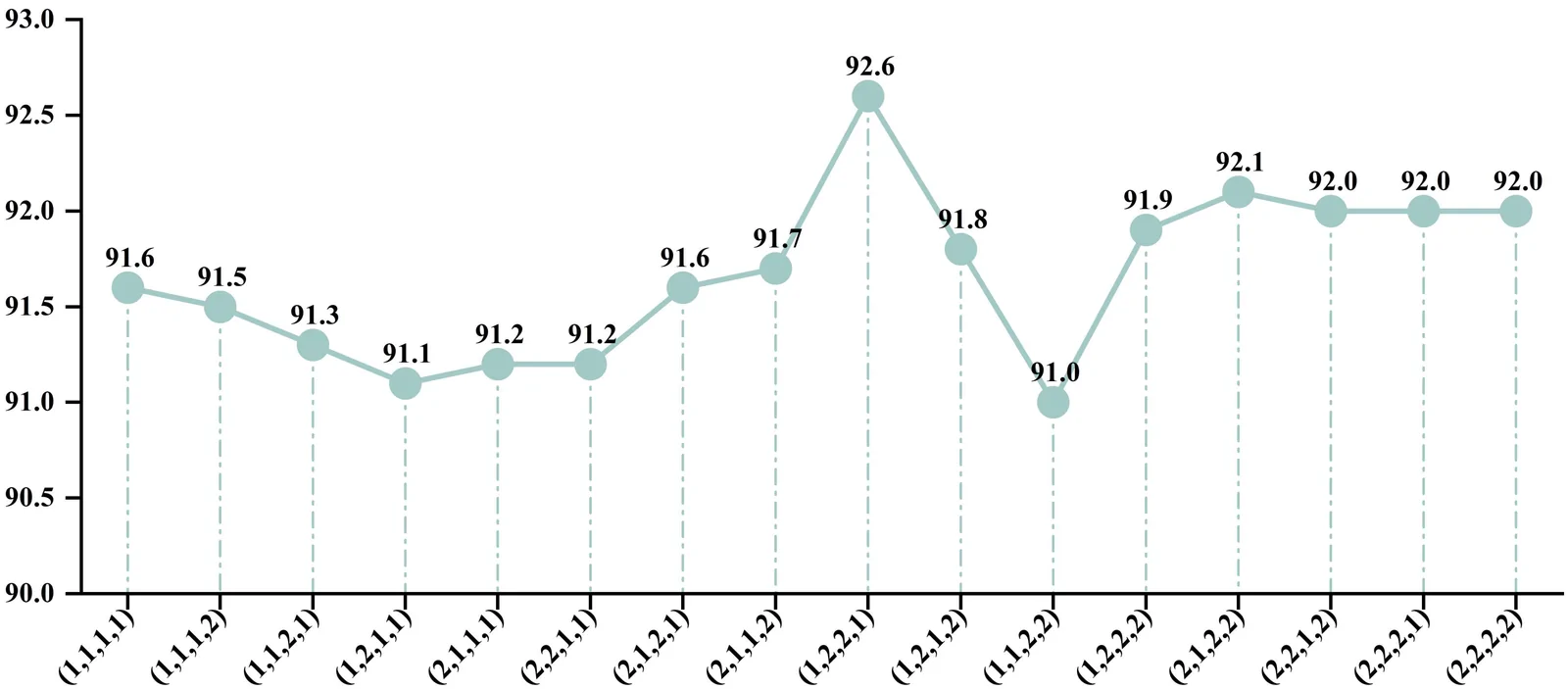
The mAP@0.5 detection results under different dilated rates. (%).

In this study, after incorporating GC modules into the Stage2-Stage4 of the backbone network, we evaluated 16 distinct configurations by systematically varying the dilation rates of the 3×3 convolutions in the Stage1-Stage4 units from (1,1,1,1) to (2,2,2,2). Since disease detection in practical applications demands high accuracy, we evaluated the mAP@0.5 metric of each configuration on the validation set. From the curves in **Figure 8**, the configuration (1,2,2,1) yields the highest mAP@0.5. Consequently, this set of rates was selected for our model, meaning all 3×3 convolutions in the Stage2 and Stage3 were set with a dilation rate of 2. That is, our final improved ResNet-50 not only replaced Bottleneck1 and Bottleneck2 with Bottleneck1_GC and Bottleneck2_GC in Stage2 to Stage4, but also adjusted the dilation rate of the 3×3 convolutions in Stage2 and Stage3 to 2.

### 3.6 Ablation experiments

To quantify the contribution of each individual component, we carried out a sequential ablation study by gradually integrating the GC (Module A), altering the dilation rate as (1,2,2,1) (Module B), adopting the OHEM strategy (Module C), and applying multi-scale training (Module D). All models were evaluated on the validation set to ensure fair comparisons. All results are reported as the mean ± standard deviation over three different random seeds (42, 123, and 456). The outcomes are summarized in **Table 4**.

**Table 4:** Ablation experiment results.

Models	Modules	mAP (%)	mAP @0.5 (%)	mAP @0.75 (%)	AR (%)	AP_S_ (%)	AP_M_ (%)	AP_L_ (%)	AP@0.5 (%)				
									Frog eye leaf spot	Powdery mildew	Rust	Scab	Mosaic
Cascade R-CNN	–	62.2 ± 0.3	91.1 ± 0.2	67.8 ± 0.7	68.8 ± 0.4	40.7 ± 0.4	37.1 ± 0.1	63.0 ± 1.5	87.5 ± 0.3	91.3 ± 0.5	86.3 ± 0.1	95.3 ± 0.3	94.8 ± 0.6
Cascade R-CNN	A	63.2 ± 0.2	92.0 ± 0.3	68.4 ± 0.9	69.3 ± 0.1	41.1 ± 0.2	36.9 ± 0.2	63.2 ± 2.1	87.5 ± 0.8	94.4 ± 1.0	87.9 ± 0.6	96.9 ± 0.9	93.3 ± 2.1
Cascade R-CNN	B	62.3 ± 0.3	91.3 ± 0.2	68.0 ± 0.7	69.0 ± 0.4	40.9 ± 0.2	41.5 ± 6.5	63.8 ± 0.9	88.1 ± 0.1	91.8 ± 0.2	86.4 ± 0.4	95.2 ± 0.7	94.8 ± 0.3
Cascade R-CNN	C	62.3 ± 0.2	90.9 ± 0.1	67.9 ± 0.3	68.9 ± 0.2	41.0 ± 0.4	38.0 ± 1.5	63.2 ± 0.3	87.2 ± 0.7	91.6 ± 0.4	86.4 ± 0.5	94.4 ± 0.6	94.9 ± 0.4
Cascade R-CNN	D	63.0 ± 0.1	92.0 ± 0.1	68.3 ± 0.4	70.0 ± 0.3	43.5 ± 0.3	38.1 ± 0.3	64.8 ± 3.0	90.3 ± 0.3	93.5 ± 0.4	88.0 ± 0.5	95.6 ± 0.2	92.3 ± 0.6
Cascade R-CNN	A+B	63.3 ± 0.3	92.2 ± 0.5	68.7 ± 0.9	69.3 ± 0.2	41.1 ± 0.4	44.1 ± 2.7	64.8 ± 1.6	88.9 ± 0.6	94.3 ± 0.6	87.2 ± 1.3	96.5 ± 0.1	94.2 ± 1.2
Cascade R-CNN	A+B+C	63.5 ± 0.3	92.4 ± 1.1	68.3 ± 1.0	69.5 ± 0.3	41.0 ± 0.5	39.9 ± 2.7	64.1 ± 0.8	88.5 ± 0.8	94.4 ± 0.9	87.0 ± 0.7	97.4 ± 0.7	94.7 ± 3.5
ALSDet	A+B+C+D	63.8 ± 0.6	92.1 ± 0.4	69.8 ± 0.9	69.8 ± 0.6	42.8 ± 0.4	38.2 ± 1.1	60.1 ± 4.4	88.9 ± 0.7	94.2 ± 1.0	87.2 ± 0.7	96.9 ± 0.5	93.1 ± 0.0

As documented in **Table 4**, the baseline Cascade R-CNN yields an mAP of 62.2%, an mAP@0.5 of 91.1%, and an mAP@0.75 of 67.8%. The combination of all four modules achieves the highest mAP, reaching 63.8%, which outperforms the baseline by a margin of 1.6%. However, the improvements come with distinct characteristics across different modules and object scales. Examining the individual contributions, Module A improves the overall mAP from 62.2% to 63.2%, corresponding to a 1.0 percentage point gain. This result demonstrates that modeling long-range channel dependencies effectively enhances the discriminative power of extracted features, enabling the network to better capture subtle lesion patterns against complex leaf backgrounds. After adopting Module B, the overall mAP rises slightly to 62.3%, while the medium-object average precision AP_M_ increases from 37.1% to 41.5%. This indicates that the optimized receptive field scheme better matches the scale distribution of medium-sized apple leaf lesions, thus improving localization accuracy for such targets. Introducing Module C brings a marginal mAP improvement of 0.1 percentage point over the baseline. The limited gain suggests that most lesion samples in our dataset have relatively distinguishable visual features. Applying Module D achieves an appreciable single-module performance boost, with mAP increasing by 0.8 percentage point to 63.0%. In particular, the small-object average precision AP_S_ rises from 40.7% to 43.5%, verifying that multi-scale training effectively strengthens the model’s ability to detect tiny lesion targets by enriching scale diversity during training.

When combining Modules A and B, the model achieves an mAP of 63.3%, and AP_M_ further increases to 44.1%, reflecting a complementary effect between global context enhancement and receptive field optimization. On this basis, adding Module C continuously improves the mAP to 63.5%, indicating that hard example mining can provide auxiliary performance gains under the multi-module framework. The full model integrating all four modules, i.e., the proposed ALSDet, achieves the best overall performance with 63.8% mAP, 92.1% mAP@0.5, and 69.8% mAP@0.75, which is 1.6 percentage points higher in mAP than the baseline Cascade R-CNN. In summary, each module promotes detection performance from different dimensions: feature representation enhancement, receptive field optimization, hard sample mining, and multi-scale adaptability. Their organic combination achieves complementary advantages and yields the optimal detection effect on apple leaf disease detection.

We further investigated the class-wise performance of each module on five specific disease categories, as shown in the last five columns of **Table 4**. Notably, the contributions of each module exhibit strong category-dependent preferences. Module A delivers relatively favorable performance gains on powdery mildew and scab, suggesting that global contextual information is particularly beneficial for diseases with regular shapes that require holistic semantic reasoning. However, it causes a slight degradation on mosaic, indicating that the introduced global features may interfere with locally dominant patterns. Module D brings substantial improvements to frog eye leaf spot and rust, which are likely characterized by large scale variations or small instances. Conversely, it also hurts mosaic, implying that multi-scale augmentation may over-regularize the model for categories with stable and salient appearances. Interestingly, mosaic suffers from performance drops across all individual modules and their combinations, with the lowest AP@0.5 reaching 92.3% in the D-only variant. This phenomenon suggests an inherent incompatibility between the feature representations of this category and our proposed modifications, which warrants further investigation. When all modules are combined, the model achieves robust performance on most categories, maintaining or slightly improving over the baseline on frog eye leaf spot, powdery mildew, rust, and scab. However, the degradation on mosaic persists, highlighting a trade-off between overall mAP gains and category-wise robustness.

### 3.7 Comparison with recent detectors

To comprehensively validate the superiority of the proposed ALSDet, we conducted comparative experiments with the mainstream object detection models on the test set, including Cascade R-CNN, Faster R-CNN (Ren et al., 2017), GFL (Generalized Focal Loss) (Li et al., 2020), Grid R-CNN (Lu et al., 2019), Libra R-CNN (Pang et al., 2019), FCOS (fully convolutional one-stage) (Tian et al., 2019), VFNet (VarifocalNet) (Zhang et al., 2021), RetinaNet (Lin et al., 2017), SSD (Liu et al., 2016), YOLOv7 (Wang et al., 2023), and YOLOv8 (Varghese and Sambath, 2024). To ensure statistical reliability and reproducibility, all experiments were repeated three times with fixed random seeds. All quantitative results are reported in the form of mean ± standard deviation. All models were trained and evaluated using exactly the same train, validation, and test split to ensure fair comparison.

As summarized in **Table 5**, the proposed ALSDet achieves the highest overall detection accuracy among all compared methods, with an mAP of 65.6% and an AR of 71.2%. Compared with the Cascade R-CNN baseline, ALSDet improves mAP by 1.3 percentage points, and outperforms the second-best GFL by 1.9 percentage points. In general, two-stage detectors exhibit better detection performance on this apple leaf disease dataset than one-stage and YOLO-series methods, mainly owing to their more accurate proposal generation mechanism and cascade bounding box regression strategy. Specifically, ALSDet surpasses the classic Faster R-CNN by 4.2 percentage points in mAP, and outperforms the representative lightweight detector YOLOv8 by 3.7 percentage points, demonstrating remarkable accuracy advantages.

**Table 5:** Performance comparison of different object detection networks.

Network model	Backbone network	mAP (%)	AR (%)	FLOPs (G)	Parameters (MB)	AP@0.5 (%)				
						Frog eye leaf spot	Powdery mildew	Rust	Scab	Mosaic
Cascade R-CNN	ResNet-50-FPN	64.3 ± 0.3	70.5 ± 0.2	182.27	41.36	88.7 ± 0.7	97.3 ± 1.3	85.6 ± 0.7	93.9 ± 0.1	90.7 ± 0.3
Faster R-CNN	ResNet-50-FPN	61.4 ± 0.2	68.4 ± 0.1	182.27	41.36	90.7 ± 0.9	96.3 ± 0.7	87.7 ± 0.3	93.5 ± 0.2	92.1 ± 1.3
GFL	ResNet-50-FPN	63.7 ± 0.5	71.3 ± 0.5	81.84	32.04	90.2 ± 1.0	96.7 ± 0.4	85.9 ± 0.6	95.2 ± 0.5	93.1 ± 0.7
Grid R-CNN	ResNet-50-FPN	61.2 ± 1.4	68.5 ± 1.4	204.49	64.25	90.2 ± 0.9	95.4 ± 0.6	87.3 ± 1.2	94.8 ± 0.1	92.2 ± 0.8
Libra R-CNN	ResNet-50-FPN	61.8 ± 0.4	68.9 ± 0.4	188	41.63	90.1 ± 0.1	94.9 ± 0.5	86.9 ± 0.1	93.6 ± 0.5	91.7 ± 0.8
FCOS	ResNet-50-FPN	60.8 ± 0.4	68.7 ± 0.3	171	32.12	89.3 ± 1.0	93.8 ± 3.1	85.4 ± 1.4	93.9 ± 0.3	92.5 ± 0.4
VFNet	ResNet-50-FPN	54.1 ± 6.3	66.5 ± 1.8	75.63	32.49	85.9 ± 2.7	80.4 ± 17.7	82.9 ± 2.3	84.9 ± 8.9	88.1 ± 2.8
RetinaNet	ResNet-50-FPN	60.6 ± 0.2	68.3 ± 0.2	179	36.41	90.5 ± 0.1	94.3 ± 0.7	85.4 ± 0.7	96.0 ± 0.2	93.5 ± 0.8
SSD	SSDVGG	54.0 ± 1.0	62.2 ± 0.8	137.97	24.28	80.6 ± 1.7	96.3 ± 0.5	79.7 ± 2.8	94.7 ± 0.5	91.5 ± 2.8
YOLOv7	YOLOv7 Backbone	48.4 ± 1.0	49.1 ± 9.9	6.56	6.02	78.9 ± 1.2	87.4 ± 1.6	79.2 ± 1.3	93.5 ± 0.3	85.2 ± 1.4
YOLOv8	YOLOv8 CSPDarknet	61.9 ± 0.2	54.9 ± 0.3	14.28	11.13	83.9 ± 0.5	93.9 ± 1.2	81.5 ± 0.6	92.4 ± 0.4	92.9 ± 1.3
ALSDet	Improved ResNet-50-FPN	65.6 ± 0.2	71.2 ± 0.2	210	71.68	89.2 ± 0.7	98.7 ± 1.0	87.3 ± 0.1	94.5 ± 1.3	93.5 ± 0.2

From the perspective of computational complexity, ALSDet has 210 GFLOPs and 71.68 MB parameters, which is moderately higher than the vanilla Cascade R-CNN baseline. This extra overhead mainly comes from the embedded GC module and the enhanced feature extraction structure. Despite the increased computational cost, ALSDet achieves the best overall performance, and such accuracy-efficiency trade-off is fully acceptable for agricultural disease detection scenarios that do not require extreme real-time inference. Notably, compared with Grid R-CNN which has similar complexity, ALSDet achieves a 4.4 percentage point higher mAP, fully verifying the effectiveness of the proposed module design.

At the category level, ALSDet achieves the best AP@0.5 on powdery mildew among all compared methods, and maintains competitive detection performance on the other four disease categories. For diseases with distinct texture features such as scab and mosaic, the detection accuracy remains at the leading tier. For challenging small-spot diseases including frog eye leaf spot and rust, ALSDet also achieves AP@0.5 scores of 89.2% and 87.3%, respectively. This performance is comparable to that of the top-performing counterparts, which further confirms that the multi-scale training and global context enhancement strategies effectively alleviate the detection bottleneck of subtle lesions. In addition, the standard deviations of all metrics are kept within a narrow range, indicating that ALSDet has stable detection performance and strong robustness under different random initializations.

Overall, the proposed ALSDet achieves the best overall performance on the apple leaf disease detection task, with outstanding detection accuracy, good category balance and reliable stability, which can well satisfy the practical demand for fine-grained identification of multiple apple leaf diseases. Furthermore, statistical significance was verified via independent two-tailed Welch’s t-test between the baseline Cascade R-CNN and the proposed ALSDet, which is suitable for small samples with potentially unequal variances. Experimental results demonstrate that ALSDet outperforms the baseline, achieving an mAP of 65.6% ± 0.2% against 64.3% ± 0.3%, with t = 6.25, df = 3.49, and p< 0.01. The Cohen’s d effect size is 5.1, indicating a very large magnitude of improvement. This finding verifies that the performance gain brought by the proposed modules is not caused by random fluctuation, and the detection accuracy improvement is statistically reliable.

### 3.8 Visualization analysis

**Figure 9:**
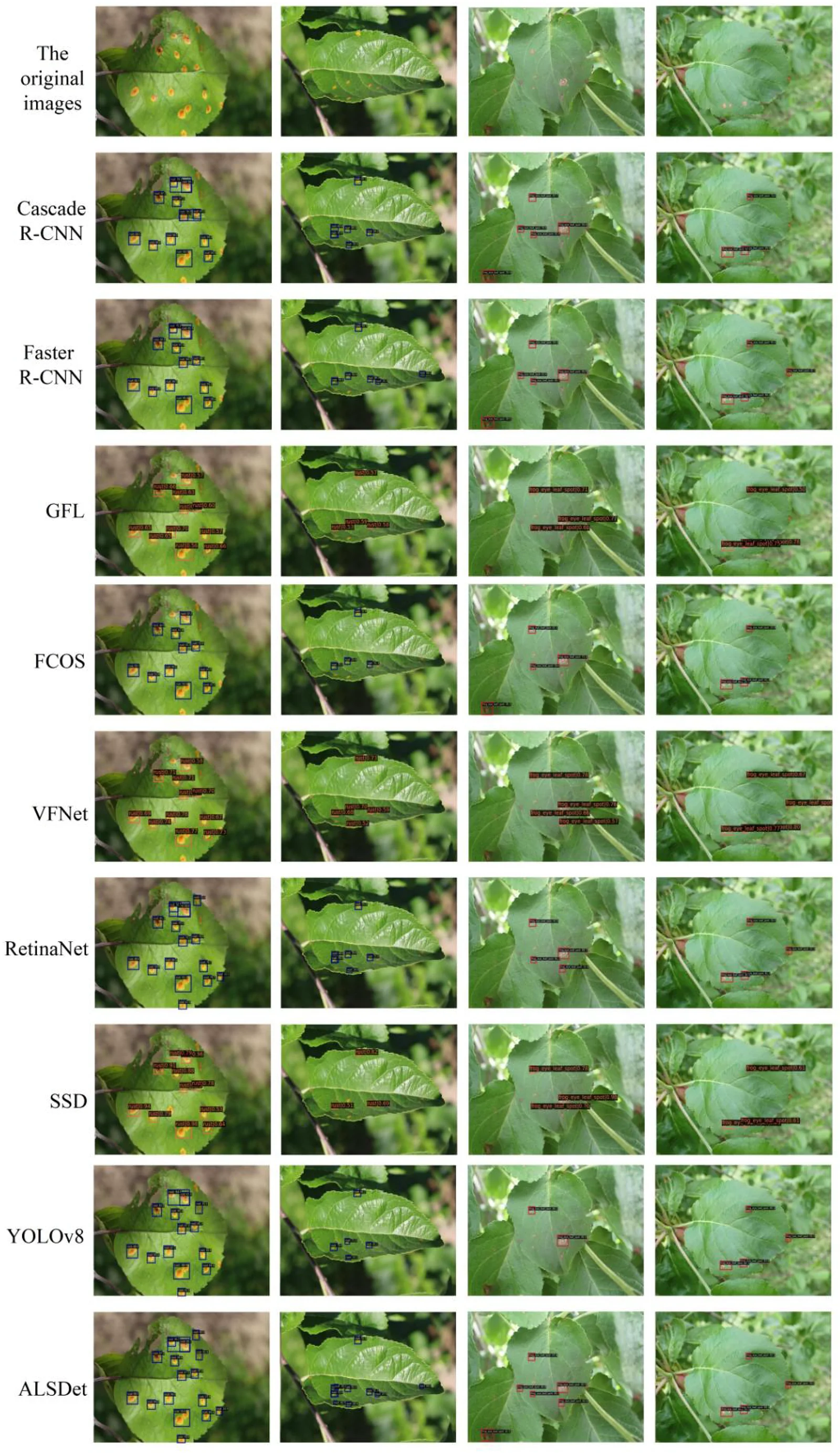
Detection performance comparison of different models.

To verify the performance of ALSDet in detecting apple leaf diseases, the proposed algorithm was compared with the networks introduced in Section 3.7 on the test dataset. The visualization results are presented in **Figure 9**. For brevity, we only present the detection results of models with imperfect predictions on these samples, while retaining the results of our proposed ALSDet as the reference baseline. As we can see from the figure, for the first and second rust-infected leaf images containing lesions of varying sizes and locations, only ALSDet successfully detects all rust lesions, while other methods exhibit missed detections. For the third image, Grid R-CNN, Libra R-CNN, YOLOv7, and ALSDet detect all frog eye leaf spot lesions. In the fourth leaf image, the detectors, excluding Cascade R-CNN, GFL, FCOS and SSD, are able to complete the identification of all frog eye leaf spot. In summary, the proposed method ALSDet not only detects apple leaf disease with higher localization accuracy, but also exhibits lower rates of missed and false detections. Particularly, it outperforms other models in detecting small targets and dense diseases.

### 3.9 Performance analysis of ALSDet

To further elucidate the recognition performance and limitations of the proposed ALSDet model across various apple leaf diseases, we conducted a fine-grained analysis from three perspectives: confusion matrix, precision-recall (PR) curves, and representative error samples.

**Figure 10:**
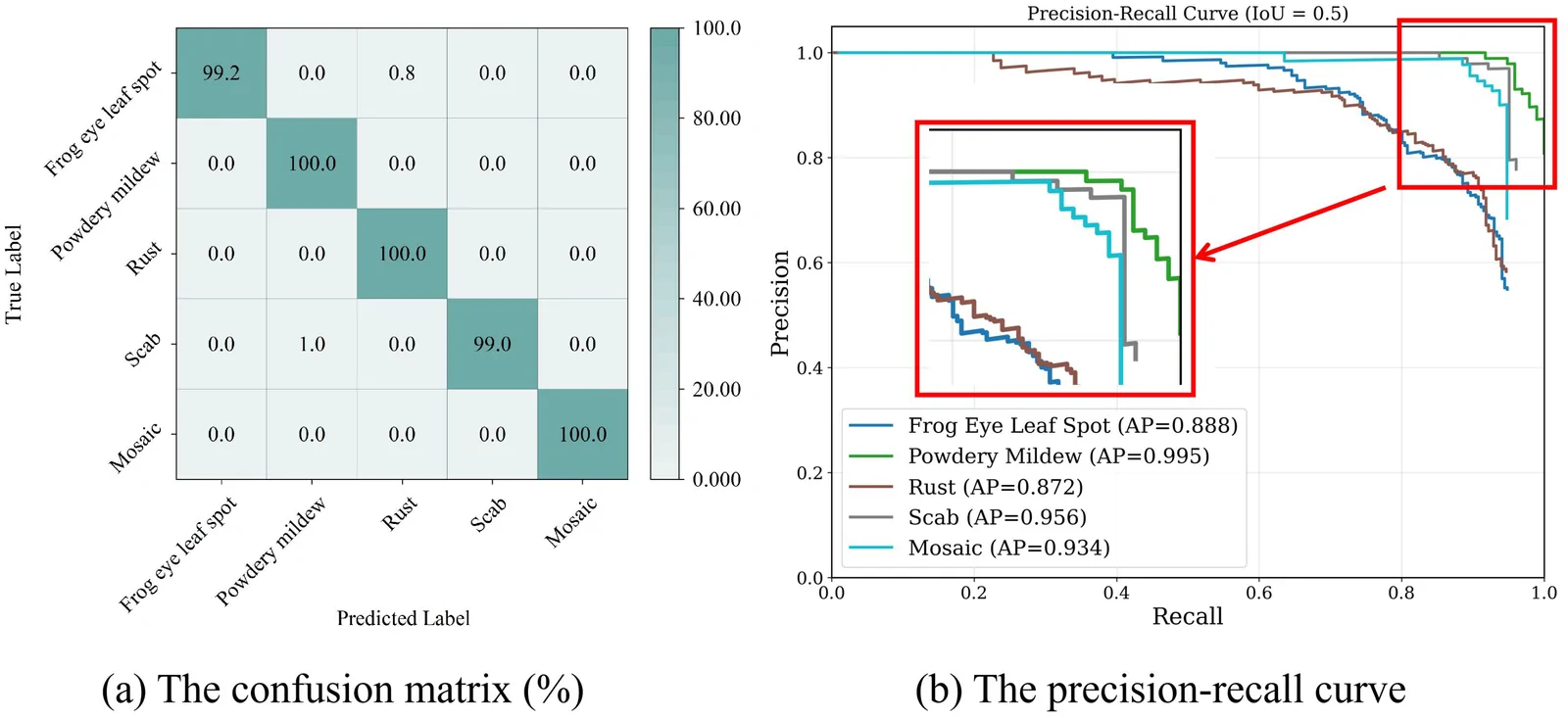
The confusion matrix and precision-recall curve of ALSDet. **(a)** The confusion matrix (%); **(b)** The precision-recall curve.

**Figure 10A** presents the normalized confusion matrix of ALSDet on the test set. Overall, the model achieves high recognition accuracy for all five disease categories. Specifically, powdery mildew, rust, and mosaic attain a recognition accuracy of 100%, indicating strong feature discriminability for these three disease types. Slight inter-class confusion is observed between frog eye leaf spot and scab: 0.8% of frog eye leaf spot instances are misclassified as rust, and 1.0% of scab instances are misclassified as powdery mildew. This confusion pattern is consistent with the visual similarity among diseases: both frog eye leaf spot and rust manifest as brown spotted lesions, which tend to produce feature overlap when lesions are small or have blurred boundaries. Besides, scab and powdery mildew both form light-colored mildew-like lesions on the leaf surface, sharing certain similarities in texture features. In general, the degree of inter-class confusion remains low, demonstrating favorable robustness in class discrimination of the proposed model.

**Figure 10B** illustrates the PR curves of each disease category at the IoU threshold of 0.5, with corresponding average precision values as follows: frog eye leaf spot 0.888, powdery mildew 0.995, rust 0.872, scab 0.956, and mosaic 0.934. As can be observed from the curve morphology, the PR curves of powdery mildew and scab lie closest to the top-right corner, maintaining high precision even in the high-recall regime. This indicates that the model yields stable detection confidence and few false positive samples for these two categories. In contrast, frog eye leaf spot and rust exhibit relatively lower AP values, with their curves descending more steeply, precision drops notably when recall exceeds 0.4. The primary reason is that lesions of these two diseases are smaller in scale and diverse in morphology, and some low-confidence detection boxes are included under high-recall settings, resulting in precision degradation. The performance of mosaic is moderate, benefiting from its typical mosaic symptoms with strong global texture features, which leads to moderate recognition difficulty for the model.

**Figure 11:**
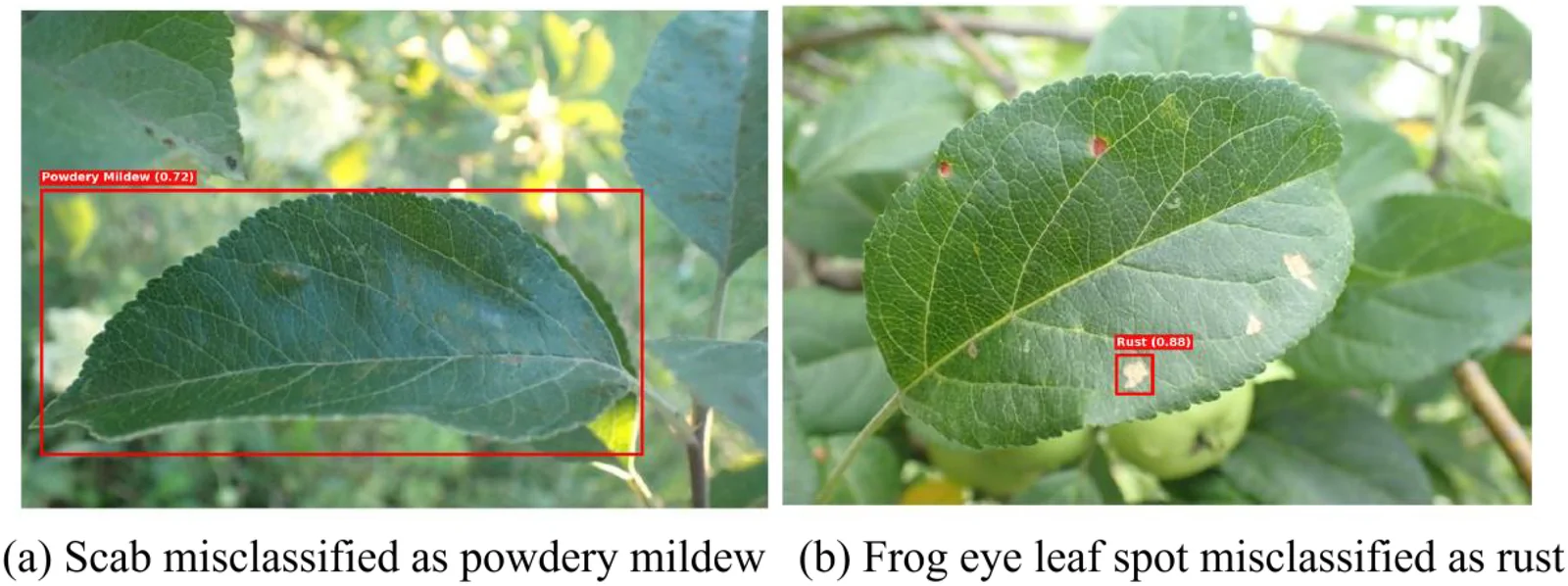
The misclassified images. **(a)** Scab misclassified as powdery mildew; **(b)** Frog eye leaf spot misclassified as rust.

**Figure 11** presents two representative detection error cases. **Figure 11A** shows a large-scale false detection case: the model misidentifies the entire leaf region as powdery mildew. This error arises from the natural gloss and light reflection on the leaf surface forming light-colored textures similar to powdery mildew, which easily triggers false detection under uneven background illumination. **Figure 11B** demonstrates a missed detection accompanied by misclassification: among multiple rust lesions on the leaf, only one is correctly detected, while the others are missed due to excessively small scale. Due to variations in shooting angles and illumination conditions, frog eye leaf spot lesions bear strong visual resemblance to rust lesions in color. Coupled with the small size of both lesion types, the detection model is prone to misclassifying frog eye leaf spot as rust.

In summary, ALSDet performs excellently on most disease categories, yet exhibits performance bottlenecks in scenarios involving similar lesions and illumination interference, which points out the direction for further algorithm improvement.

## 4 Discussion

In response to the challenges posed by small-target apple leaf diseases in complex natural environments, we developed ALSDet. This framework includes the Cascade R-CNN detection pipeline, ResNet-50-FPN backbone, GC block, dilated convolution, OHEM and multi-scale training. None of these single components represents absolute original inventions in this work. Instead, the novelty lies in how we adaptively integrate these mature components to tackle the specific challenge of detecting tiny lesions in complex orchard backgrounds.

First, we optimize the feature extraction backbone to alleviate feature degradation of small early lesions in deep convolutional networks. In field-collected apple leaf images, tiny disease targets easily lose discriminative structural features and blend into background clutter after repeated downsampling operations in the backbone. The GC module is embedded in the middle and high stages of the ResNet backbone to model long-range channel-wise dependencies, which enhances the semantic discriminability of lesion features against complex leaf backgrounds. The optimized dilation rate configuration adjusts the backbone’s receptive field distribution without additional downsampling, recovering partial spatial resolution lost during deep feature encoding. This adjustment is highly matched to the scale distribution of medium-sized apple leaf lesions. Together, these two feature-level improvements effectively preserve fine-grained contour details of micro lesions while enhancing feature representation capability.

Second, two targeted training strategies are introduced to further boost the model’s robustness under unconstrained orchard imaging conditions. The OHEM strategy focuses the model on hard negative samples with textures similar to real lesions during training. Additionally, multi-scale training enriches the scale diversity of training samples and enhances the model’s scale invariance. It delivers the most prominent single-module improvement for small targets, and effectively improves the detection accuracy of small-target diseases under variable shooting distances in actual orchards. In summary, the four modules optimize detection performance from four dimensions: feature representation, receptive field matching, hard sample classification, and scale adaptability. Their complementary effects jointly achieve the optimal comprehensive detection performance on the apple leaf disease dataset.

However, there are limitations to our work that need to be considered. First, the dataset contains only five common apple leaf diseases, excluding other diseases and insect pests. Furthermore, it consists exclusively of optical images as the single type of image data, preventing the model from learning comprehensive spectral and structural features of diseased leaves. The current work does not include external validation across independent orchards and growing seasons, which limits the full verification of the model’s cross-scene generalization ability. Second, the proposed model has a relatively large parameter volume and computational footprint, making it difficult to achieve efficient and stable operation when deployed on mobile devices or embedded devices.

To overcome the aforementioned shortcomings, our future efforts will be directed toward two key directions:(1) Multi-modal data enhancement: We will broaden the data collection to increase the types of diseases and the number of samples, carry out systematic external validation, and further optimize the model’s cross-domain generalization performance for practical orchard deployment. Furthermore, we will organically integrate multi-modal data from various sensors to furnish more comprehensive and reliable information for detecting apple leaf diseases. (2) Model lightweighting and deployment: We will minimize the model’s computational complexity and memory footprint as much as possible while ensuring detection accuracy, thereby improving the model’s operational efficiency on mobile devices. These advancements are expected to enhance the practical utility of ALSDet. By providing reliable decision-support to growers and enabling seamless integration into broader agricultural Internet of Things (IoT) platforms, this technology can facilitate automated monitoring and timely interventions, thereby promoting more sustainable and precision-oriented orchard management practices.

## 5 Conclusion

The proposed model, ALSDet, achieves competitive performance in identifying small lesion targets on apple leaves within complex natural scenarios. Based on the Cascade R-CNN framework, ALSDet incorporates the ResNet-50 backbone enhanced with the GC module and dilated convolution, alongside OHEM and multi-scale training strategies, to improve feature representation and model robustness.

Through systematic experimentation, we determine the optimal network configuration, which involves inserting the GC module into the Stage2 to Stage4 and applying a dilation rate of 2 to the 3×3 convolutions in Stage2 and Stage3. Ablation studies confirm the individual and collective contributions of these components to the overall performance. Besides, OHEM and multi-scale training are employed. The final model achieves an mAP of 65.6% and an AR of 71.2%, demonstrating the effectiveness of these improvements. In comparative experiments, ALSDet outperform other mainstream detection models, exhibiting superior accuracy and robustness. Notably, it achieves high AP scores of 87.3% for rust and 89.2% for frog eye leaf spot, validating its precision in identifying specific small-target diseases.

The proposed ALSDet delivers competitive detection accuracy with moderate computational overhead, providing a viable technical solution for automated apple leaf disease monitoring in natural orchard environments. Future work will focus on integrating this technology into mobile or edge computing devices for real-time field applications and extending the model to identify a broader spectrum of plant diseases.

## Data Availability

The raw data supporting the conclusions of this article will be made available by the authors, without undue reservation.
